# Neuro-ophthalmologic and blood biomarker responses in ADHD following subconcussive head impacts: a case–control trial

**DOI:** 10.3389/fpsyt.2023.1230463

**Published:** 2023-11-22

**Authors:** Madeleine K. Nowak, William G. Kronenberger, Devin Rettke, Osamudiamen Ogbeide, Lillian M. Klemsz, Patrick D. Quinn, Timothy D. Mickleborough, Sharlene D. Newman, Keisuke Kawata

**Affiliations:** ^1^National Center for PTSD at VA Boston Healthcare System, Boston, MA, United States; ^2^Department of Psychiatry, Boston University Chobanian & Avedisian School of Medicine, Boston, MA, United States; ^3^Department of Kinesiology, School of Public Health-Bloomington, Indiana University, Bloomington, IN, United States; ^4^Department of Psychiatry, Indiana University School of Medicine, Indianapolis, IN, United States; ^5^Department of Applied Health Science, School of Public Health-Bloomington, Indiana University, Bloomington, IN, United States; ^6^Program in Neuroscience, College of Arts and Sciences, Indiana University, Bloomington, IN, United States; ^7^Alabama Life Research Institute, University of Alabama, Tuscaloosa, AL, United States

**Keywords:** ADHD, neuro-ophthalmologic function, blood biomarkers, subconcussion, concussion, traumatic brain injury

## Abstract

**Introduction:**

This clinical trial aimed to determine the influence of attention-deficit/hyperactivity disorder (ADHD) on neuro-ophthalmologic function and brain-derived blood biomarkers following acute subconcussive head impacts.

**Methods:**

The present trial consisted of age- and sex-matched samples with a ratio of 1:1 between two groups with a total sample size of 60 adults (age ± SD; 20.0 ± 1.8 years). Soccer players diagnosed with and medicated daily for ADHD were assigned into an ADHD group (*n* = 30). Soccer players without ADHD were assigned into a non-ADHD group (*n* = 30). Participants performed 10 soccer headers with a soccer ball projected at a velocity of 25mph. King-Devick test (KDT), near point of convergence (NPC), and serum levels of NF-L, tau, GFAP, and UCH-L1 were assessed at baseline (pre-heading) and at 2 h and 24 h post-heading.

**Results:**

There were no statistically significant group-by-time interactions in outcome measures. However, at baseline, the ADHD group exhibited lower neuro-ophthalmologic functions compared to the non-ADHD group (NPC: *p* = 0.019; KDT: *p* = 0.018), and persisted at 2 h-post (NPC: *p* = 0.007; KDT: *p* = 0.014) and 24 h-post heading (NPC: *p* = 0.001). NPC significantly worsened over time in both groups compared to baseline [ADHD: 2 h-post, 1.23 cm, 95%CI:(0.77, 1.69), *p* < 0.001; 24 h-post, 1.68 cm, 95%CI:(1.22, 2.13), *p* = 0.001; Non-ADHD: 2 h-post, 0.96 cm, 95%CI:(0.50, 1.42), *p* < 0.001; 24 h-post, 1.09 cm, 95%CI:(0.63, 1.55), *p* < 0.001]. Conversely, improvements in KDT time compared to baseline occurred at 2 h-post in the non-ADHD group [−1.32 s, 95%CI:(−2.55, −0.09), *p* = 0.04] and at 24 h-post in both groups [ADHD: −4.66 s, 95%CI:(−5.89, −3.43), *p* < 0.001; Non-ADHD: −3.46 s, 95%CI:(−4.69, −2.23), *p* < 0.001)]. There were no group-by-time interactions for GFAP as both groups exhibited increased levels at 2 h-post [ADHD: 7.75 pg./mL, 95%CI:(1.41, 14.10), *p* = 0.019; Non-ADHD: 7.91 pg./mL, 95%CI:(1.71, 14.14), *p* = 0.015)] that returned to baseline at 24 h-post. NF-L levels increased at 2 h-post heading in the ADHD group [0.45 pg./mL, 95%CI:(0.05, 0.86), *p* = 0.032], but no significant NF-L changes were observed in the non-ADHD group over time.

**Discussion:**

Ten soccer headers elevated GFAP levels and NPC impairment in both groups. However, persisting group difference in NPC, blunted KDT performance, and increased NF-L levels in the ADHD group suggest that ADHD may reduce neuro-ophthalmologic function and heighten axonal response to soccer headers.

**Clinical trial registration:**

ClinicalTrials.gov, identifier ID: (NCT04880304).

## Introduction

Research focusing on subconcussive head impacts has increased exponentially in the past two decades (1). The current consensus is that even mild head impacts, if sustained repetitively, can cause impairments in eye movement (2), elevations in neural-injury blood biomarkers (3, 4), and changes in neuronal network (5). With chronicity, these neurological alterations can be amplified and may predispose individuals to later onset of neurodegenerative conditions (1, 6). Several lines of research have begun suggesting a few demographic factors that may modulate neural resiliency to subconcussive head impacts (7). Specifically, attention-deficit/hyperactivity disorder (ADHD) has been shown to reduce resiliency to trauma induced brain injury (8).

ADHD is the most common neurodevelopmental disorder (9), and as many as 14% of high school and 10% of college athletes are reported to be diagnosed with ADHD (10). The majority of these athletes participate in contact/collision-prone sports (e.g., American football, soccer, hockey) (11). Growing evidence indicates that athletes with ADHD have a 1.5- to 5-fold increased risk for concussion (10, 12), which is accompanied by increases in repeated prevalence (13) and in symptom severity and time to recovery compared to athletes without ADHD (10). Furthermore, ADHD has recently been suggested to increase vulnerability to subconcussive head impacts, as exhibited by acute declines in cognitive performance and increases in blood biomarker levels associated with neuronal injury (ubiquitin-C-terminal-hydrolase-L1; UCH-L1) and astrocyte activation (glial-fibrillary-acidic-protein; GFAP) following 10 acute soccer headers (8). These preliminary findings suggest that ADHD-specific characteristics, such as delayed maturation of prefrontal cortex (14) and dopaminergic neural pathways (15) and reduced cortical thickness and brain volume (16), may influence outcomes of brain trauma.

Unlike subjective measures such as self-reported symptoms, neuro-ophthalmologic function has shown to be one of the most sensitive functional metrics to reflect the severity of neural stress from subconcussive head impacts (17). Neuro-ophthalmologic function integrates signals from visual, oculomotor, and cognitive inputs. For example, near point of convergence (NPC) (2, 18) measures the closest point of focus before diplopia occurs, and the King-Devick test (KDT) (2) couples saccadic eye movements with multiple facets of brain functions, such as attention, language, and concentration (19). Our recent clinical trial found that 10 soccer headings significantly impaired neuro-ophthalmologic functions in soccer players without ADHD (2). The use of brain-derived blood biomarkers has also proven sensitive to objectively assess neural integrity following brain injury (4). In particular, tau, neurofilament light polypeptide (NF-L), glial-fibrillary-acidic protein (GFAP), and ubiquitin-C-terminal hydrolase-L1 (UCH-L1) have shown to gauge the effects of concussive (20) and subconcussive head impacts (3, 8). Increased plasma NF-L expression was observed in college-aged soccer players following 10 soccer headers and was able to distinguish from the kicking-control group (3). Similar utility of blood biomarkers has been seen in soccer players diagnosed with ADHD (8). However, the findings of previous studies (8, 10) that identified ADHD as an antecedent risk factor for brain injury were limited by small sample size, limited diagnostic assessment for ADHD, and non-demographically matched controls.

Therefore, to address these limitations, we designed a clinical trial using our soccer heading model (21) and a multi-modal approach consisting of neuro-ophthalmologic function and the brain-derived blood biomarkers (NF-L, Tau, UCH-L1, GFAP) to examine the role of ADHD in repetitive subconcussive head impacts. Our primary hypothesis was that soccer players with ADHD would demonstrate a greater neurological distress to subconcussive head impacts, as reflected in decreased NPC and KDT performance and in a higher magnitude of increased serum levels of blood biomarkers at post-heading time points compared to soccer players without ADHD.

## Materials and methods

### Participants

**Figure 1:**
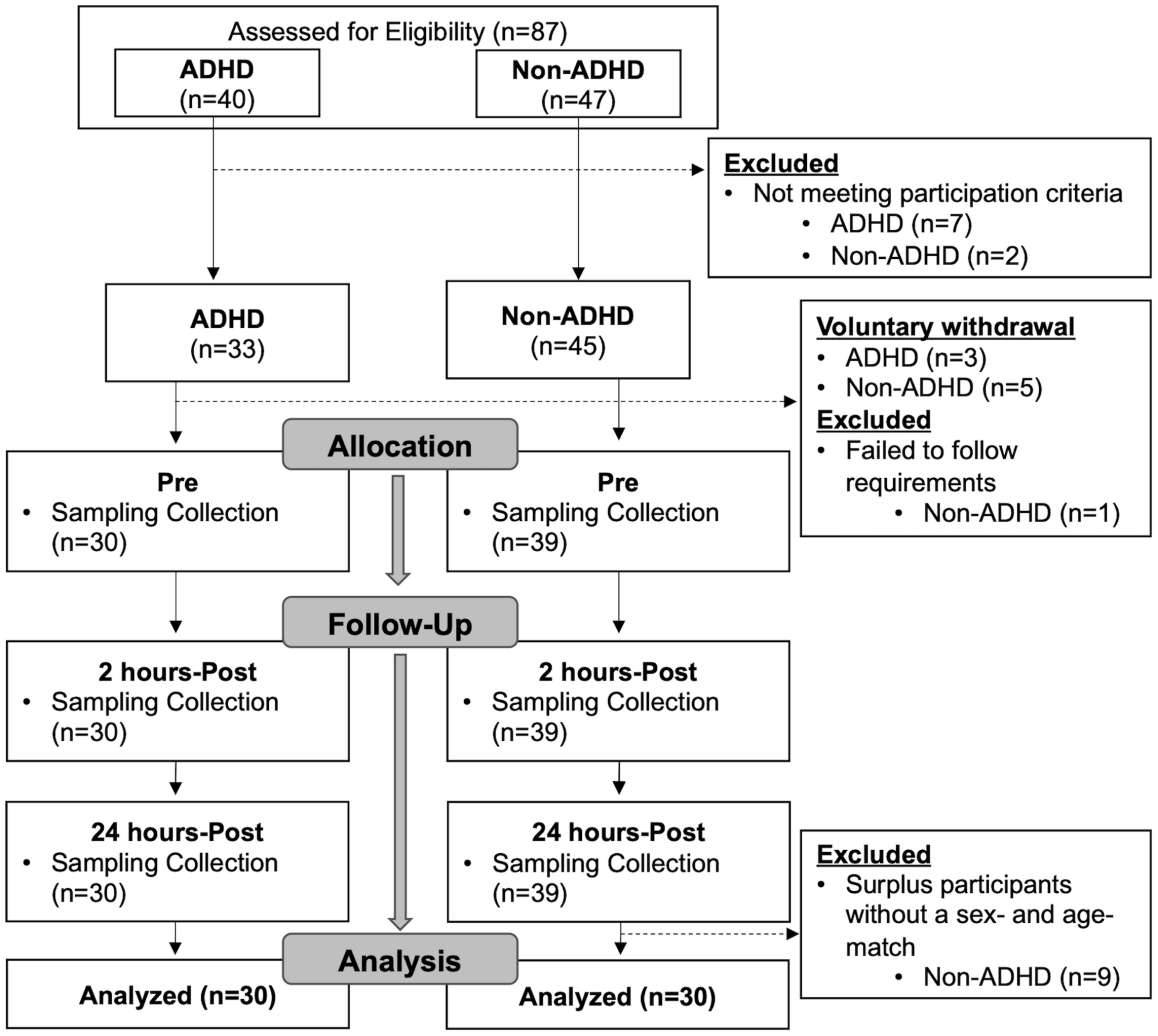
Study flow chart.

From March 2021 through March 2022, potential participants were simultaneously recruited from local universities and soccer clubs, and 87 potential participants were screened to be assigned into one of two groups: (1) individuals clinically diagnosed with and medicated for ADHD (ADHD group) and (2) age- and sex-matched individuals without ADHD diagnosis (non-ADHD group). For any non-ADHD participants whose age and sex demographics were not matched in the ADHD group, they continued to participate in the study but were not included in the analysis. As a result, a total of 69 participants completed the study and 60 were included in the final analysis (ADHD group, *n* = 30; non-ADHD group, *n* = 30; Figure 1). Inclusion criteria across both groups consisted of being between the ages of 18 and 26 years and having at least 5 years of soccer heading experience. Participants in the ADHD group were also required to be taking their prescribed ADHD medication at least 5 days a week. The non-ADHD group had no prior or current history of taking ADHD medication. Exclusion criteria for both groups included a head or neck injury including concussion with symptoms remaining within 3 months prior to the start of the study, and any current or prior history of learning disabilities (e.g., dyslexia, processing deficits) or major mental disorders (e.g., schizophrenia, bipolar disorder, autism spectrum disorder) other than ADHD. Participants who reported a current or history of anxiety or depressive disorder were included in the study, as long as symptoms were not quantified as severe in their psychiatric assessment or by their provided psychological diagnostic reports (22) (see Diagnostic Assessment for ADHD and Psychiatric Factors section). This criterion was identified prior to the start of recruitment and was included in our initial assessment to preserve generalizability due to the increasing prevalence of anxiety and major depressive disorders diagnosed in college-aged individuals (23), athletes (24), and as a result of the COVID-19 Pandemic (25).

### Trial design

Following recruitment, a self-reported health questionnaire was administrated to each participant to obtain demographic information, head impact history, and mental health conditions. Serum samples were collected, and participants performed NPC and KDT at three collection time points: [pre-heading baseline, 2 h-, and 24 h-post soccer heading]. Following baseline measurements, participants performed 10 controlled soccer headers (see Subconcussion Model section). Participants remained in the laboratory until the 2 h-post heading time point. Participants returned to the laboratory approximately 24 h after the soccer heading for the final time point. The post soccer heading timepoints have been used previously in similar study designs and have been shown to be a sufficient timeframe to identify the neuro-ophthalmologic and cellular responses to subconcussive head impacts (2, 8). From 3 days prior to the start of the study and continuing until the 24 h post-heading time point, participants were instructed to (1) take their prescribed ADHD medication (for the ADHD group), (2) refrain from consuming alcohol and recreational drugs, and (3) refrain from activities that involve head impacts. If participants failed to comply with the instructions, they were either rescheduled or withdrawn from the study. The study protocol was approved by the Indiana University Institutional Review Board and was registered under ClinicalTrials.gov (NCT04880304). All participants provided written informed consent.

### Diagnostic assessment for ADHD and psychiatric factors

To confirm ADHD diagnosis and to assess ADHD symptoms, a well-trained research coordinator, who was advised by two licensed psychologists with expertise in ADHD, conducted a semi-structured diagnostic interview using the Adult ADHD Investigator Symptom Rating Scale (AISRS) (26). Two additional trained testers assessed the agreement of AISRS total symptom scores. Absolute inter-rater reliability was excellent (intraclass correlation coefficient, 0.99 [95% CI, 0.97 to 1.00]; *p* < 0.001). The coordinator and testers were blinded to status of the participants’ ADHD diagnosis. In compliance with the AISRS, participants in the ADHD group were required to report at least five symptoms from the symptom-checklist of the Diagnostic and Statistical Manual of Mental Disorders 5^th^ Edition (DSM-5) in either the inattentive or the hyperactive/impulsive domain and were free of symptoms unrelated to ADHD (i.e., schizophrenia, substance withdrawal). Participants in the non-ADHD group were required to score below the AISRS threshold. Further validation for the ADHD group included documentation of their complete clinical ADHD diagnosis. Additional psychiatric assessments included the Beck Anxiety Inventory (BAI) (27), Patient Health Questionnaire (PHQ-9) (28), General Anxiety Disorder (GAD-7, 27) Alcohol Use Disorder Identification Test (AUDIT) (29), Cannabis Use Disorder Identification Test (CUDIT-R) (30), and Daily Stress Inventory (DSI) (31).

### Subconcussion model

A soccer heading model was used to induce subconcussive head impacts (2, 8, 21). Several of our prior studies have validated this model to provide adequate force to induce subconcussive injury, as seen by elevated neural injury biomarkers and impairments in neuro-ophthalmologic, oculomotor, and cognitive functions following repeated exposure to soccer headings (2, 3, 8). See Bevilacqua et al. for the video version (21). A triaxial accelerometer (GFroceTracker Inc., Ontario, CA) was used to measure head accelerations. A JUGS soccer machine (JUGS Sports, Tualatin, OR) was used to launch a size-5 soccer ball at a traveling velocity of 25 mph (11.2 m/s), which is a similar speed to a long throw-in and on the slower-scale end of rising balls kicked by adult soccer players (32), to participants who stood approximately 40 ft. (12.2 m) away from the JUGS machine. Participants were instructed to use the standard method of hitting the ball with the center of their forehead to perform 10 headers within 1-min intervals to a targeted individual who stood about 16 ft. (4.9 m) from participants.

### Neuro-ophthalmologic assessment

Assessment consisted of previously established protocols of NPC and KDT (2). NPC was measured using the accommodative ruler (Gulden Ophthalmics, Elkins Park, PA) and was taken when participants verbally signaled diplopia had occurred or the researcher observed misalignment. Neuro-ophthalmologic functional integrity, using saccadic eye movement and cognitive processing speed, were examined with KDT on a tablet. Participants performed saccades while rapidly reading numbers aloud and their total time (seconds) and total errors were recorded.

### Blood biomarker assessment

Six millimeters of venous blood were collected into vacutainer tubes (BD Biosciences, San Jose, CA) at pre (baseline), 2 h-, and 24 h-post soccer heading for blood biomarker analysis. A selective sample were not valid for venipuncture methodology due to participant anxiety or phlebotomy difficulty (*n* = 5). In place of venipuncture, a non-invasive Tasso sampling kit (Tasso inc.) was placed on participants’ arms and used to collect a total of 400–600 uL of capillary blood per device. Clotted serum was centrifugated (1,500 x *g*, 15 min, 4 °C), aliquoted, and stored at-80 °C prior to running the assay. Serum levels of NF-L, Tau, UCH-L1, and GFAP were simultaneously ran over three assays using the Human Neurology 4-Plex A assay (N4PA) on a SR-X with single molecule array (Simoa^™^, Quanterix) (33). Samples were loaded in duplicate according to Quanterix instructions. To eliminate the inter-assay effect on within-subject data, all samples from each participant were ran on the same plate. Limit of detection (LOD) was 0.136 pg./ mL for NF-L, 0.276 pg./mL for GFAP, 0.0298 pg./mL for Tau, and 4.03 pg./mL for UCH-L1.

### Primary and secondary outcome measures

To address if ADHD is a modulating factor for subconcussive head impacts and account for differences between groups at baseline, the primary interest was to test the changes in KDT time and error, NPC distance, and in serum levels of NF-L, Tau, UCH-L1, and GFAP over time between the ADHD and non-ADHD groups (group-by-time interaction). Our secondary interest was to test the changes in neuro-ophthalmologic function and serum levels over time compared to baseline values within the ADHD group.

### Statistical analysis

The assessment of demographic differences between the ADHD and non-ADHD groups at baseline consisted of student’s two-tailed independent t-tests for continuous variables (age, BMI, number of previous concussion, years of soccer experience, years of soccer heading experience, age started soccer, hours of sleep, SCAT symptom scores, AUDIT scores, CUDIT scores) and Fisher’s exact tests for categorical variables (sex, race, ethnicity, diagnosis of additional mental disorder, history of COVID-19). Independent samples t-tests were also used to compare baseline values of the outcome variables between groups. A series of mixed-effects regression models was used to test our hypotheses. The primary (fixed-effect) factors were groups (ADHD and non-ADHD), time of measurement (pre-heading baseline, 2 h-, and 24 h-post heading), and the group-by-time interaction. Participants were treated as a random effect to account for individual differences at baseline. A sensitivity analysis was conducted to evaluate whether any demographic variables, regardless of group difference, were significantly associated with the outcome measures. Methods of outlier detection were calculated to examine unusual influence of individual data points on the fit of the model. Additional validation of Tasso measurements was used and data points that were identified as outliers were excluded from the group analysis. Following the removal of appropriate Tasso samples, six data points were identified as outliers (GFAP: *n* = 0, NF-L: *n* = 4, Tau: *n* = 1, UCH-L1: *n* = 1). Nine data points for UCH-L1 were below the LOD, and 5 samples were not valid for analysis due to phlebotomy or SR-X complications. The model was refit without these data being included. For each estimated outcome value (β coefficient), a 95% confident interval (CI) was calculated, and missing data was accounted for. The analysis was summarized by providing a contrast estimate with its 95% CI and a value of *p* in the following format: (estimate [95% CI, low CI-CI high], value of *p*). The significance levels for all tests were set *a priori* to 0.05. Lastly, a post-hoc analysis of three independent t-tests (NPC, KDT time, KDT error) each with a Bonferroni adjusted alpha level of 0.016 was conducted to examine group differences in neuro-ophthalmologic function at each post-heading time point. All analyses were conducted using statistical software R version 3.5.2 (R Foundation for Statistical Computing) with packages “lmer” and “lmerTest.”

## Results

### Demographics

Eighty-seven individuals were assessed for eligibility, and 78 individuals who met the inclusion criteria, and were free of exclusion criteria, proceeded to the study. Changes in schedules and COVID-19 exposure/diagnosis attributed to eight voluntary withdrawals prior to the study data collection, and one individual was withdrawn due to failure to comply with study instructions. Nine participants who were free of ADHD diagnosis were unable to be age- and sex-matched to participants in the ADHD group and were excluded from the analysis. As a result, data from 60 participants were valid for analysis: ADHD, *n* = 30 and non-ADHD, *n* = 30 (Figure 1). Demographics and head impact kinematics are presented in Table 1. Significant differences in number of previous concussion and diagnosis of additional mental disorder were observed and were accounted for by the mixed-effects regression models.

**Table 1:** Demographics and head impact kinematics by group.

Variables	ADHD	Non-ADHD
*n*	30	30
Sex	13 M 17\u00B0F	13 M 17\u00B0F
Age, *y*	19.9 ± 1.88	20.1 ± 1.83
BMI, *kg/m^2^*	23.1 ± 5.20	24.3 ± 3.59
No. of previous concussion**		
0, *n (%)*	14 (46.7)	25 (83.3)
1, *n (%)*	13 (43.3)	5 (16.7)
2, *n (%)*	3 (10)	0 (0)
Soccer Experience, *y*	11.1 ± 3.64	12.4 ± 3.02
Soccer Heading Experience, *y*	7.8 ± 2.46	9.1 ± 2.89
Race, *n (%)*		
White	24 (80)	24 (80)
Black/African American	3 (10)	2 (6.7)
Asian	1 (3.3)	4 (13.3)
More than one race	2 (6.7)	0 (0)
Ethnicity, *n (%)*		
Not Latino/Hispanic	28 (93.3)	28 (93.3)
Latino/Hispanic	2 (6.7)	2 (6.7)
ADHD Medication, *days per week*	6.4 ± 0.89	^a^
Psychiatric Assessment, *total no. of symptoms*		
AISRS: Inattention	7.4 ± 1.45	0.3 ± 0.66
AISRS: Hyperactivity-Impulsivity	5.2 ± 2.33	0.3 ± 0.70
SCAT5, *baseline*	3.2 ± 3.06	2.2 ± 2.52
AUDIT	5.7 ± 4.27	6.1 ± 4.13
CUDIT	4.8 ± 6.95	2.5 ± 4.19
Additional Mental Disorder Diagnosis, n (%)***	17 (56.7)	4 (13.3)
Prior COVID-19 Infection, *n (%)*	10 (33.3)	10 (33.3)
Head Impact Kinematics per Header		
PLA, *g*	13.6 ± 1.98	13.9 ± 2.45
PRA, *(°/s^2^)*	859.6 ± 198.7	822.2 ± 248.2
Baseline Serum Blood Biomarker Levels, *pg/mL*		
Glial Fibrillary acidic protein (GFAP)	66.1 ± 26.16	70.9 ± 32.68
Neurofilament light (NF-L)	5.3 ± 2.85	5.0 ± 2.37
Tau	0.85 ± 0.36	0.67 ± 0.40
Ubiquitin C-terminal hydrolase-L1 (UCH-L1)	13.2 ± 14.40	9.1 ± 5.00
Neuro-ophthalmologic Performance		
Near Point of Convergence (NPC), cm*	7.0 ± 2.03	5.8 ± 1.89
King-Devick Test (KDT) time, s*	51.5 ± 9.59	46.0 ± 7.9
King-Devick Test (KDT) error, *total^#^*	0.4 ± 0.72	0.3 ± 0.65

All data is reported as group means of each demographic ± standard deviations, unless otherwise specified. BMI: body mass index. AISRS: Adult ADHD Investigator Symptom Rating Scale. SCAT5: Sport Concussion Assessment Tool-5. AUDIT: Alcohol Use Disorders Identification Test. CUDIT: Cannabis Use Disorders Identification Test. PLA: peak linear acceleration. PRA: peak rotational acceleration. ^a^Individuals were free from ADHD diagnosis and had no history of taking ADHD medication. Asterisk (*) refers to statistically significant differences between groups at baseline: *p < 0.05; **p < 0.01; ***p < 0.001. Significant differences between groups at baseline were seen in previous concussion (*p* = 0.002) and in additional mental disorder diagnosis (*p* < 0.001), and in baseline neuro-ophthalmologic performance metrics of NPC (*p* = 0.019) and KDT time (*p* = 0.018).

### Subconcussive effects on neuro-ophthalmologic function

**Figure 2:**
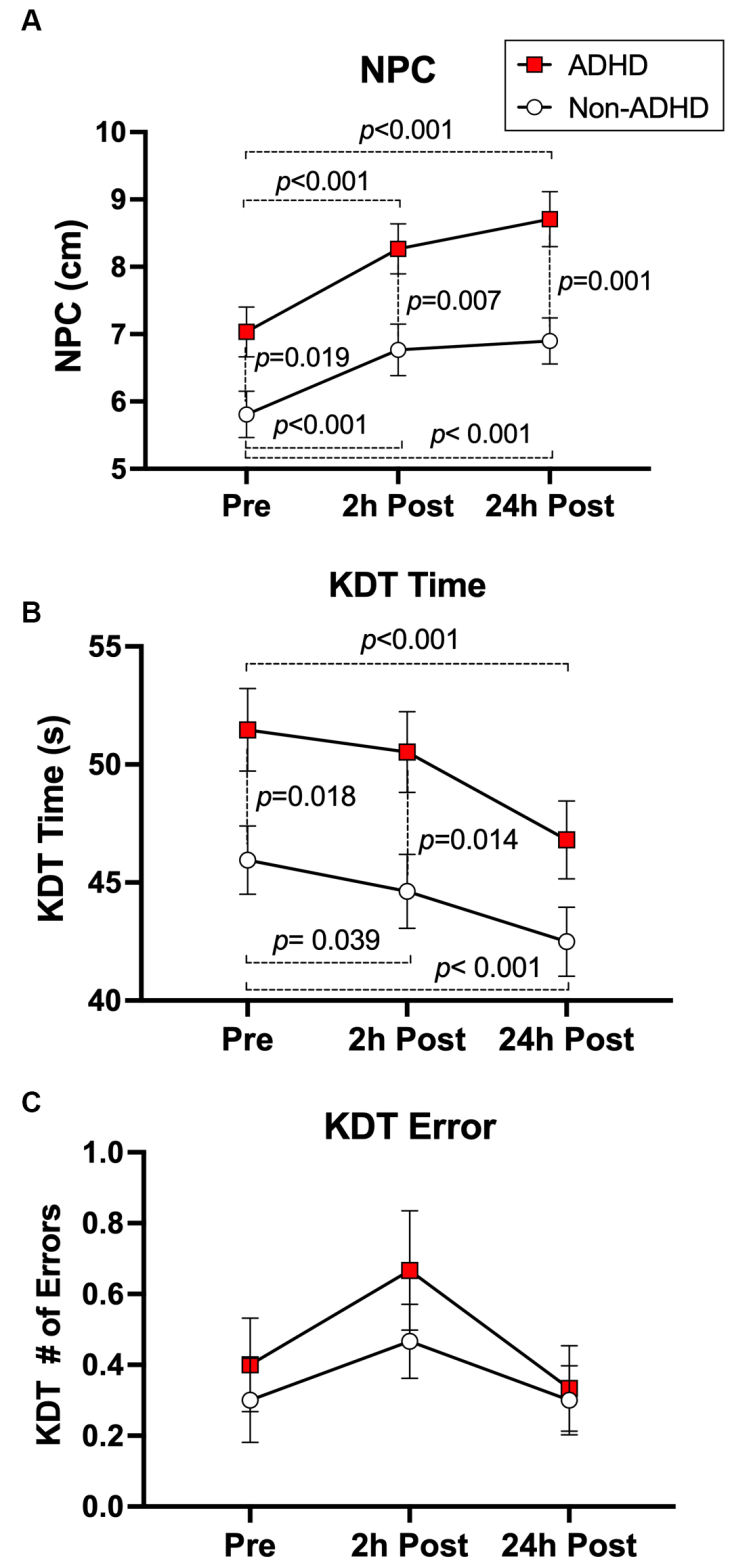
Subconcussive effects on **(A)** NPC, **(B)** KDT time, and **(C)** KDT error. There were no group-by-time interactions in neuro-ophthalmologic function following 10 soccer headers. Ten soccer headers blunted the neuro-ophthalmologic ability to learn and adapt to KDT time in the ADHD group at 2 h-post heading, whereas a significant improvement in time was seen in the non-ADHD group. Subconcussive head impacts significantly worsened NPC for up to 24 h in both groups, whereas KDT time improved at 24 h and KDT error remained unchanged. The ADHD group performed significantly worse than the non-ADHD group at baseline in NPC (*p* = 0.019) and in KDT time (*p* = 0.018). A *post-hoc* analysis with an adjusted alpha level (α = 0.016) revealed that the ADHD group consistently performed worse than the non-ADHD group in NPC at all post-heading time points, and in KDT time at 2 h-post heading. Values graphed represent group means at each time points and the standard error of the mean (SEM).

Ten soccer headers significantly increased (worsened) NPC in both groups compared to their pre-heading baselines (ADHD: 2 h-post, 1.23 cm [0.77, 1.69], *p* < 0.001; 24 h-post, 1.68 cm [1.22, 2.13], *p* < 0.001; Non-ADHD: 2 h-post 0.96 cm [0.50, 1.42], *p* < 0.001; 24 h-post 1.09 cm [0.63, 1.55], *p* < 0.001; Figure 2A). The ADHD group displayed worse NPC at baseline compared to the non-ADHD group (*p* = 0.02), and the group difference persisted at 2 h (*p* = 0.007) and 24 h-post heading (*p* = 0.001). However, there were no group-by-time interactions at 2 h- or 24 h-post heading.

Despite sustaining 10 soccer headings, both groups displayed improvements in KDT time at 24 h-post compared to their pre-heading baseline (ADHD: −4.66 s [−5.89, −3.43], *p* < 0.001. Non-ADHD: −3.46 s [−4.69, −2.23], *p* < 0.001; Figure 2B). The non-ADHD group also improved KDT time at 2 h-post compared to baseline (−1.32 s [−2.55, −0.09], *p* = 0.04), whereas the ADHD group showed no changes at 2 h-post compared to baseline (−0.94 s [−2.17, 0.29], *p* = 0.14). However, the group-by-time interaction was not statistically significant at either time point (Table 2). Slower KDT time was seen in the ADHD group compared to the non-ADHD group at baseline (*p* = 0.018), as well as at 2 h-post heading (*p* = 0.014); however, the group difference disappeared at 24 h-post heading. No significant changes were observed in KDT error (Figure 2C). Within-group changes from baseline for neuro-ophthalmologic metrics are presented in Table 3. Between-group results are presented in Table 4.

**Table 2:** Group-by-time interaction in serum blood biomarker concentrations and neuro-ophthalmologic function.

Biomarker	2 h-post	*p* value	24 h-post	*p* value
GFAP	0.16 [−8.72 to 9.05]	0.972	6.45 [−2.30 to 15.19]	0.155
NF-L	−0.31 [−0.87 to 0.25]	0.288	−0.10 [−0.66 to 0.44]	0.712
Tau	−0.14 [−0.30 to 0.02]	0.136	−0.08 [−0.23 to 0.08]	0.038*
UCH-L1	−0.52 [−4.47 to 3.43]	0.801	0.30 [−3.63 to 4.19]	0.884
NPC, *(cm)*	−0.28 [−0.93 to 0.38]	0.413	−0.58 [−1.23 to 0.07]	0.084
KDT time, *(s)*	−0.38 [−2.11 to 1.36]	0.675	0.07 [−0.53 to 2.94]	0.180
KDT error, *(#)*	−0.10 [−0.52 to 0.32]	0.650	0.07 [−0.36 to 0.49]	0.762

Between-group estimates (β) with their 95% confidence intervals (CI) for our primary interests of changes in biomarkers over time between the ADHD and non-ADHD groups. A group-by-time interaction in tau was observed at 24 h-post heading. The blood biomarkers are measured in units of pg/mL. Asterisk (*) refers to statistically significant group-by-time interaction between the ADHD and non-ADHD groups: *p < 0.05.

**Table 3:** Within-group changes in neuro-ophthalmologic function from baseline.

Assessment	Group	2 h-post	*p* value	24 h-post	*p* value
NPC *(cm)*	ADHD	1.23 [0.77 to 1.69]	<0.001***	1.68 [1.22 to 2.13]	<0.001***
	Non-ADHD	0.96 [0.50 to 1.42]	<0.001***	1.09 [0.63 to 1.55]	<0.001***
KDT time *(s)*	ADHD	−0.94 [−2.17 to 0.29]	0.139	−4.66 [−5.89 to-3.43]	<0.001***
	Non-ADHD	−1.32 [−2.55 to-0.09]	0.039*	−3.46 [−4.69 to-2.23]	<0.001***
KDT error *(#)*	ADHD	0.27 [−0.33 to 0.57]	0.088	−0.07 [−0.37 to 0.23]	0.668
	Non-ADHD	0.00 [−0.13 to 0.47]	0.285	0.16 [−0.30 to 0.30]	1.000

Within-group estimates (β) with their 95% confidence intervals (CI) for each post-heading timepoint in reference to variables at pre-heading baseline. Asterisk (*) refers to statistically significant differences between post heading and baseline performance values: *p < 0.05; ***p < 0.001.

**Table 4:** Group differences in neuro-ophthalmologic function at post-heading timepoints.

Assessment	Timepoint	ADHD	Non-ADHD	*p* value
NPC *(cm)*	2 h-post	8.3 ± 2.0	6.8 ± 2.1	0.007 *
	24 h-post	8.7 ± 2.2	6.9 ± 1.9	0.001**
KDT time *(s)*	2 h-post	50.5 ± 9.4	44.6 ± 8.6	0.014*
	24 h-post	46.8 ± 9.0	42.5 ± 8.0	0.055
KDT error *(#)*	2 h-post	0.67 ± 0.9	0.47 ± 0.6	0.318
	24 h-post	0.33 ± 0.7	0.30 ± 0.5	0.831

Data are reported as group means ± standard deviations. A Bonferroni adjusted alpha level of 0.016 was used for the three post-hoc independent groups *t*-tests. Asterisk (*) refers to statistically significant differences in post-heading values between the ADHD group and the non-ADHD group: *p < 0.016; **p < 0.003. Baseline differences in neuro-ophthalmologic performance can be found in Table 1.

### Subconcussive effects on neural-injury blood biomarkers

**Figure 3:**
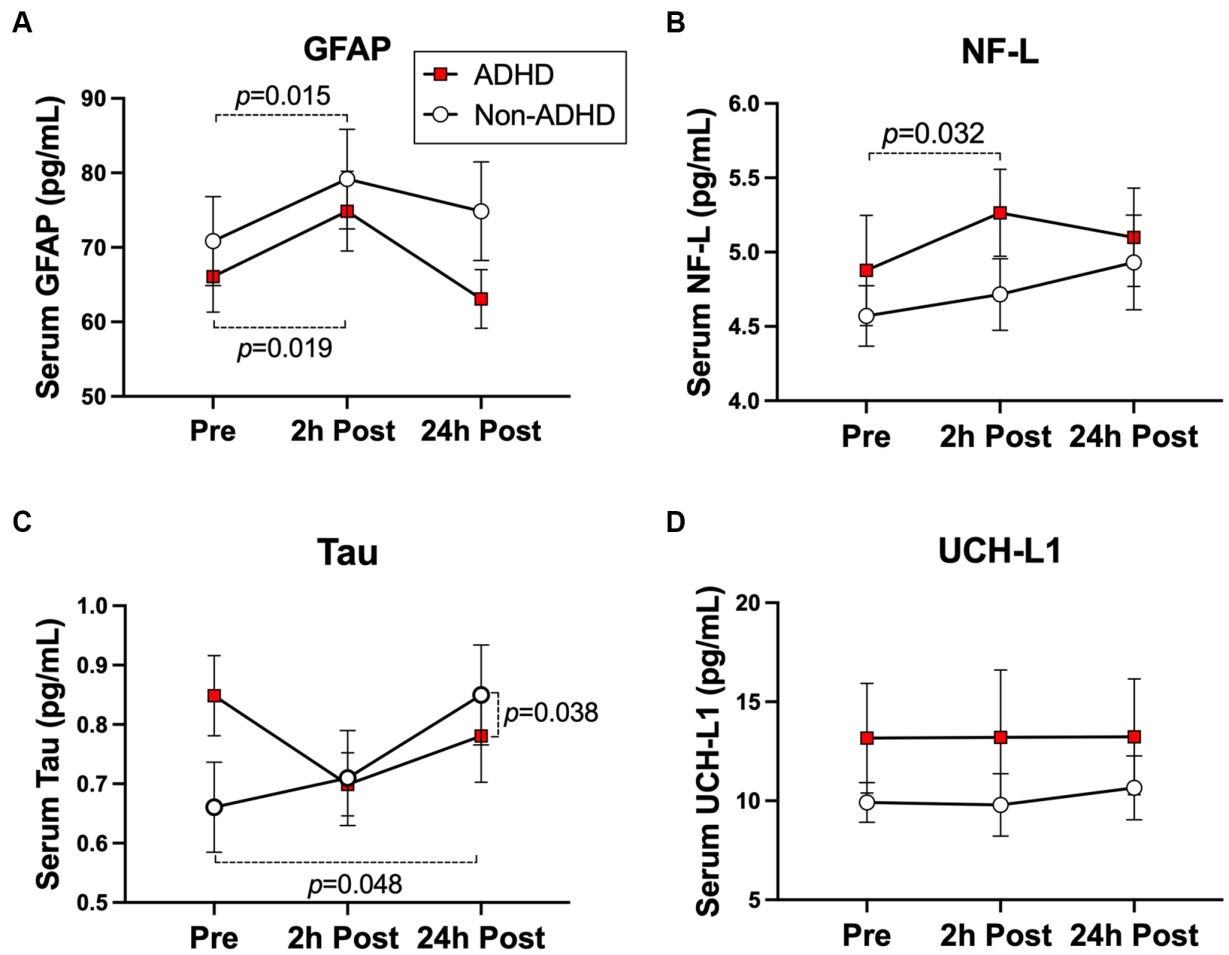
Subconcussive effects on **(A)** NF-L, **(B)** GFAP, **(C)** UCH-L1, and **(D)** Tau. Within-group changes in the ADHD group were observed in post-heading time points compared to baseline. A significant elevation in NF-L levels (*p* = 0.032) was specific to the ADHD group. However, significant elevations in GFAP levels (ADHD: *p* = 0.019, non-ADHD: *p* = 0.015) were observed regardless of ADHD status. There were no significant differences in blood serum biomarker levels between groups at baseline. Values graphed represent group means at each time point and the standard error of the mean (SEM).

The ADHD group exhibited a significant increase in NF-L levels at 2 h-post heading compared to baseline (0.45 pg./mL [0.05, 0.86 pg./mL], *p* = 0.032) but no change at 24 h-post heading (0.19 pg./mL [−0.20, 0.59 pg./mL], *p* = 0.340). Conversely, the non-ADHD group displayed no significant change across all time points (Figure 3A). Both groups displayed significant increases in GFAP levels at 2 h-post heading (ADHD: 7.75 pg./mL [1.41, 14.10 pg./mL], *p* = 0.019; Non-ADHD: 7.91 pg./mL [1.71, 14.14 pg./mL], *p* = 0.015), and GFAP levels in both groups were normalized to baseline levels at 24 h-post heading (ADHD: −2.45 pg./mL [8.67, 3.76 pg./mL], *p* = 0.446; Non-ADHD: 4.00 pg./mL [−2.16, 10.15 pg./mL], *p* = 0.210; Figure 3B). There was no significant change in UCH-L1 levels in both groups across at all time points (Figure 3C). A significant group-by-time interaction was observed in tau levels at 24 h-post heading, where the non-ADHD group exhibited higher levels of tau (0.24 pg./mL [0.02 to 0.46], *p* = 0.038; Figure 3D). Conversely, there were no group-by-time interactions for NF-L, GFAP, or UCH-L1 levels (Table 2). Within-group changes from baseline are presented in Table 5.

**Table 5:** Within-group changes in serum blood biomarker concentrations from baseline.

Blood Biomarker	Group	2 h-post	*p* value	24 h-post	*p* value
GFAP	ADHD	7.75 [1.41 to 14.10]	0.019*	−2.45 [−8.67 to 3.76]	0.446
	Non-ADHD	7.91 [1.71 to 14.14]	0.015*	4.00 [−2.16 to 10.15]	0.210
NF-L	ADHD	0.45 [0.05 to 0.86]	0.032*	0.19 [−0.20 to 0.59]	0.340
	Non-ADHD	0.14 [−0.24 to 0.53]	0.473	0.08 [−0.30 to 0.47]	0.657
Tau	ADHD	−0.14 [−0.30 to 0.02]	0.085	−0.08 [−0.23 to 0.08]	0.335
	Non-ADHD	0.03 [−0.13 to 0.19]	0.709	0.16 [0.01 to 0.32]	0.048*
UCH-L1	ADHD	0.58 [−2.20 to 3.39]	0.687	−0.002 [−2.68 to 2.69]	0.999
	Non-ADHD	0.07 [−2.77 to 3.11]	0.963	0.29 [−2.55 to 3.12]	0.841

Within-group estimates (β) with their 95% confidence intervals (CI) for each post-heading timepoint in reference to blood biomarkers at pre-heading baseline. The blood biomarkers are measured in units of pg/mL. Asterisk (*) refers to statistically significant differences between post heading and baseline values: *p < 0.05.

## Discussion

The purpose of this clinical trial was to assess whether neuro-ophthalmologic and cellular responses to acute subconcussive head impacts differ between individuals diagnosed with ADHD and those without ADHD. By strengthening the reliability of ADHD diagnosis and medication requirements, and minimizing the potential influences of sex, age, and recreational drug and alcohol use on neural-injury blood biomarkers, this trial exceeded the rigor of the previous reports and revealed that regardless of ADHD status, acute subconcussive head impacts equally impaired NPC for at least 24 h. However, ADHD participants exhibited reduced ability to improve KDT time at 2 h-post heading compared to their non-ADHD counterparts, who showed significant improvements at all post-heading time points, suggesting that ADHD may have the potential to impair neuro-ophthalmologic response to subconcussion. We also provide evidence to suggest that as mild as 10 controlled soccer headers can acutely induce astrocytic activation regardless of ADHD status, as reflected by transient elevation in GFAP levels at 2 h-post heading, and that neuronal axons may respond differently to repetitive head impacts in individuals with ADHD, as reflected by increased NF-L levels in the ADHD group only and increased tau levels in the non-ADHD group only. Taken together, these findings suggest that 10 soccer headers can trigger astrocyte activation and ocular-motor impairments in both groups. However, persisting group difference in NPC, blunted KDT performance, and increased serum NF-L levels in the ADHD group suggest that ADHD may reduce neuro-ophthalmologic function and heighten axonal response to soccer headers.

Our finding on NPC agrees with previous reports showing that NPC is a sensitive clinical measure to monitor acute subconcussive head impacts (2). Ten soccer headings significantly worsened NPC compared to baseline in the ADHD group by 17% at 2 h-post heading and by 23% at 24 h-post heading, whereas the non-ADHD group exhibited significant increases by 16% at 2 h-post heading and by over 18% at 24 h-post heading. Despite these variations, there were no significant group-by-time interactions following subconcussive head impacts. ADHD has been linked to ocular abnormalities and impairment in NPC (34, 35). For example, Ababneh et al. (35) found that children diagnosed with ADHD performed significantly worse on NPC than their siblings without ADHD. It is important to note that the NPC changes observed are likely not clinically significant. However, it is statistically significant and similar decreases in NPC were observed in a previous study that identified mild oculomotor impairment in college-aged soccer players not diagnosed with ADHD following 10 soccer headers (2). Another novel aspect of our study is that improvements in KDT time at 2 h-post heading were observed in the non-ADHD group only. The accuracy of KDT relies on recruiting a greater number of brain pathways compared to other oculomotor tests (e.g., NPC) (2). This recruitment includes prefrontal and cingulate cortices, cerebellum, corpus callosum, basal ganglia, and the occipital lobe (36). Our observed result in KDT time is plausible, given that ADHD has been shown to influence these functions and anatomical regions (14). A similar response was seen in our previous study, in that the non-ADHD group demonstrated learning effects in visual working memory despite sustaining 10 soccer headings. Contrarily, 10 headings blunted the ADHD group’s ability to learn and improve visual working memory score (8). These data substantiate the findings that ADHD may decrease resiliency to subconcussion, beyond deficits observed in non-ADHD counterparts, particularly in broad aspects of cognitive functions, such as working memory, attention, and anticipation. Although this is the first study observing repeat test taking of KDT in ADHD, the potential learning effect on KDT is identified in non-ADHD athletes free from concussion with a reported improvement of 1.2 s following a bout of 10 soccer headings (2) and of approximately 2 to 4 s across a season (37, 38). As an earlier study by Hakvoort et al. (34) demonstrating the usefulness of fMRI to identify ADHD-specific neural activation pattern during an antisaccade task, future studies should incorporate fMRI to explore the neuro-mechanistic interaction between ADHD and subconcussion. It is also worth noting that ADHD medication was unable to improve neuro-ophthalmologic performance to a level comparable to their non-ADHD counterparts at baseline. Furthermore, our post-hoc analysis indicated that despite the combination of repeat test taking and medication, the ADHD group consistently exhibited impairments in NPC at all post-heading time points and in KDT time for at least 2 h. Medication treatment has been shown to resolve neuro-ophthalmologic decrements in pediatric patients with ADHD (35); however, the neuro-ophthalmologic influence of medication on college-aged soccer players is limited to the present study.

Another novel finding our results provided is that, regardless of ADHD diagnosis, acute astrocyte activation occurs following 10 soccer headings to GFAP elevations, as reflected by similar increases in GFAP levels in the ADHD and non-ADHD group. This finding is different from our preliminary results, in which elevations in GFAP were specific to the ADHD group. Discrepancy in the two studies may be explained by a variety of factors and their influences on astrocyte integrity, cellular hypertrophy, and ultimately GFAP upregulation. First, the sample size of the current trial is approximately 2-fold higher than our previous study (8), and our non-ADHD control group is sex- and age-matched. Although data is limited, astrocyte activation has been suggested to be sex- and age-dependent, as GFAP elevations have been shown to increase with age (39) and correlate with levels of sex steroid hormones (39). Second, the method of neural-injury blood biomarker assessment varied between studies. This may play a role in the differential data, given that total GFAP concentrations have been shown to differ between serum and plasma samples from the same participants (40). However, both serum (20) and plasma (8) samples have been used successfully to identify increases in GFAP related to head injury, and to be similar in the ability to distinguish intracranial abnormalities (serum: AUC = 0.814 and plasma: AUC = 0.778) (40). Another important methodical factor to consider is the use of a semi-automated SR-X device in the present trial, rather than a fully-automated Simoa HD-1 analyzer, as used in our previous study (8). Despite the potential risk of human error using SR-X, the two devices have shown a high correlation (*R^2^* = 0.9412) (41). Third, the duration of taking ADHD medication was lengthened and included 3 days prior to the start of the study, in addition to during the course of the study as implemented in our pilot study (8). Non-toxic doses of ADHD medication has been shown to activate glial cells without significantly increasing GFAP levels (42). This is important due to the fact that moderate activation of astrocytes has shown neuroprotective effects (43). Fourth, we instructed participants to refrain from substance use to control for acute recreational drug and alcohol usage. Postmortem examination has shown that cannabis may decrease astrocyte activation (44), and that acute withdrawal from alcohol use may increase GFAP levels in rodent models (45). Ultimately, we attempted to address the limitations of our previous work and other studies (3, 46) by strengthening the reliability of ADHD diagnosis and medication requirements, and minimizing the potential influences of sex, age, and recreational drug and alcohol use on neural-injury blood biomarkers. Our study isolated the influence of ADHD on neural integrity and indicated that 10 bouts of subconcussive head impacts can increase serum GFAP levels, regardless of ADHD diagnosis.

On the contrary, our data have demonstrated that ADHD may influence the axonal response to subconcussive head impacts. Blood NF-L levels in the ADHD group were shown to be sensitive to 10 controlled soccer headers, with serum levels increasing at 2 h-post heading. Elevations in blood NF-L concentration have been established as a reliable marker to assess axonal damage following subconcussive head impacts (4) and increases have been suggested to be associated with total number of concussion (47). However, previous studies, including ours (8), have failed to properly control for ADHD diagnosis. The rigorous inclusion and exclusion ADHD diagnostic criteria used in the present study allowed us to isolate and link our observed NF-L elevations to an ADHD diagnosis. Serum NF-L is a reputable blood biomarker due to its success in assessing brain injury severity and its correlation with neuroimaging outcomes (47). For example, elevations of serum NF-L were found to be correlated with decreases in total volume and integrity of white matter in adult hockey players (47), and with cognitive deficits and brain atrophy in patients with mutations associated with Alzheimer’s Disease (48). Meta-analyses of neuroimaging data in ADHD report that similar atypical microstructural patterns (16), and decreases in neural circuitry and cerebral volume (49) are able to distinguish patients with ADHD from healthy controls without an ADHD diagnosis. In a longitudinal study of 288 children, Sudre et al. (50) implemented two measurements of DTI and fMRI and discovered that worsening symptoms of ADHD were associated with decreases in intrinsic connectivity and integrity of white matter. The analysis from the present study design suggest that these ADHD-specific characteristics may heighten the cellular response to acute subconcussive head impacts, as seen by serum NF-L elevations observed in the ADHD group only.

Despite also originating from the axon, serum tau levels opposed those of NF-L, as reflected by elevated tau levels in the non-ADHD group, and a significant group-by-time interaction, at 24 h-post heading. Tau is one of the most studied biomarkers in TBI research; however, majority of existing literature identifies acute exposure to subconcussive head impacts to be below the threshold for increased tau phosphorylation (4, 51). The tau elevations displayed in the present study propose that further investigation is warranted in order to identify other possible factors that contribute to serum tau levels. UCH-L1 is also predominately used for more severe forms of brain injury, with existing studies failing to detect UCH-L1 elevations following subconcussive head impact exposure (46). Neither of the groups in our study exhibited elevations in UCH-L1 across time points, suggesting that 10 controlled soccer headers is not sufficient to induce neuronal injury. Further research is required to disentangle the complexities in the results from our present study and our preliminary study (8).

### Clinical implication

Our data supports the present literature that neural-injury blood biomarkers, KDT and NPC have the potential to aid in athlete monitoring. However, additional measurements, such as neuroimaging, should be evaluated to determine clinical decisions. Despite the possible vulnerabilities associated with ADHD that our studies suggest, exercise and sport participation has shown to improve mental and physical health in individuals with ADHD (52). Collectively this should all be considered when monitoring safety in athletes with ADHD.

### Limitations

The present study has several limitations that should be considered. We are unable to account for additional factors that may influence our outcome variables. For example, participants were unmonitored in between time points of 2 h- and 24 h-post heading. Individual variabilities may also have an impact on the outcome variables, as NF-L has been suggested to be associated with concussion history in prior studies (47). However, our study design of repeated measures designed to compare the individual responses to baseline and our approach to treat participants as a random effect with mixed effects regression models should minimize any potential subject-level influences independent of ADHD status on the primary interests. The final models were determined with a balance of parsimony and best-fitting and potential covariates (e.g., number of previous concussion, mental health status) were included in the models to further minimize the potential influences. Despite the atypical performance of our soccer heading model of 10 consecutive soccer headers within 1-min intervals, our model allowed us to isolate ADHD and eliminate extraneous influences that are inherent in field studies. Future studies are encouraged to link the activation of astrocytes to suggested biological influencers and to address the study’s limited generalizability, including expanding data collection to larger-scale settings.

## Conclusion

The present study provides evidence that ADHD may have increased vulnerability to cellular and neuro-ophthalmologic deficits at baseline and after repetitive subconcussive head impacts. Regardless of ADHD diagnosis, subconcussive head impacts increased serum GFAP levels and impaired NPC. Conversely, further observation of ADHD-specific reduced resiliency to subconcussive-induced neural injury was reflected in increased serum NF-L levels and in blunted improvements in KDT time compared to their non-ADHD counterparts. We encourage future studies to use additional neurological measures to confirm these findings.

## Data availability statement

The raw data supporting the conclusions of this article will be made available by the authors, without undue reservation.

## Ethics statement

The studies involving humans were approved by Indiana University Institutional Review Board. The studies were conducted in accordance with the local legislation and institutional requirements. The participants provided their written informed consent to participate in this study.

## Author contributions

MN: conceptualization, collection and curation of the data, interpretation, formal analysis, writing – original draft, writing – review and editing. WK: conceptualization, interpretation, formal analysis, writing – review and editing. DR: collection and curation of the data, writing – review and editing. OO and LK: collection of the data, writing – review and editing. PQ: conceptualization, interpretation, formal analysis, writing – review and editing. TM: conceptualization, interpretation, formal analysis, writing – original draft, writing – review and editing, administration, supervision. SN: conceptualization, interpretation, writing – review and editing, funding acquisition, supervision. KK: conceptualization, interpretation, formal analysis, writing – original draft, writing – review and editing, funding acquisition, administration, supervision. All authors contributed to the article and approved the submitted version.
